# Effect of anxiety and depression levels on pregnancy outcome

**DOI:** 10.1590/1806-9282.20230922

**Published:** 2024-03-04

**Authors:** Filiz Demirhan Pinarbasi, Fatma Basar, Ahmet Fatih Oguc

**Affiliations:** 1 Fertillife Afyon Hospital Obstetrics, Gynecology and In Vitro Fertilization Center – Afyonkarahisar, Turkey.; 2 Kutahya Health Sciences University, Faculty of Health Sciences, Department of Obstetrics and Gynecology Nursing – Kütahya, Turkey.

**Keywords:** Infertility, Anxiety, Depression, Pregnancy outcome, In vitro fertilization

## Abstract

**OBJECTIVE:**

The aim of this study was to determine the effect of anxiety and depression on pregnancy outcome in couples receiving in vitro fertilization treatment.

**METHODS:**

A total of 102 couples (102 females and 102 males) with unexplained infertility were included in the study. Personal Information Form was used to collect data, Case Follow-up Form to record the treatment process, and Spielberger’s State-Trait Anxiety Inventory and Beck Depression Inventory to measure the anxiety and depression levels of couples. Couples were measured twice: before treatment and on oocyte pickup day.

**RESULTS:**

There was no statistically significant difference between the anxiety and depression levels and oocyte count of women (p>0.05). There were statistically significant differences between State-Trait Anxiety Inventory level and sperm count and between BID level and sperm motility (p<0.05). There was no statistically significant difference between the anxiety and depression levels and pregnancy outcomes of women (p>0.05).

**CONCLUSION:**

Anxiety and depression had no effect on pregnancy outcome. More studies are needed to investigate the effect of anxiety and depression on pregnancy outcome in unexplained infertility.

## INTRODUCTION

Infertility is estimated to affect 8–15% of reproductive-aged couples worldwide. The rates are different in high- and low-income countries. In some developing regions of the world, the rates of infertility are much higher, reaching 30% in some populations^1^. In Turkey, there are 1.5–2 million infertile couples^2^. Due to high prevalence of infertility, it has been highlighted as a social disease by the World Health Organization (WHO)^1^. Infertile couples experience pressure from society, family, and treatment and have a higher risk of negative emotions, such as anxiety and depression. In vitro fertilization-embryo transfer (IVF-ET) is a method in which infertile couples participate to achieve pregnancy. This method has the problems of high cost and uncertain treatment effect, which may cause couples undergoing IVF-ET to experience great pressure. IVF-ET involves different stages, including ovulation induction, ovulation retrieval, and embryo transfer, and patients will face various difficulties in different stages. For example, patients in the stage of ovulation induction will worry about the effect of ovulation-promoting drugs, pain, the number of oocytes taken before ovulation, and whether the embryo can be implanted after transplantation. Whether the anxiety and depression of infertile couples in diverse stages of IVF-ET are different and whether they are related to the outcome of assisted pregnancy have been rarely reported. Turkish infertile couples may be affected by the traditional idea of succession, and they are under greater psychological pressure^3^. Women who experience infertility report elevated levels of stress, anxiety, and depression. However, the impact of depressed mood and anxiety on infertility treatment outcome remains unclear^1,4,5^. Therefore, further investigations in this area are necessary.

Most of the studies on infertility in our country and worldwide have been conducted on women. Studies in which couples are evaluated together are quite limited. However, it is not only women who are affected by infertility. In addition, when the studies investigating the relationship between infertility and psychological factors are examined, it is seen that these studies are mostly in the form of comparing the fertile population with the infertile population or determining the psychological factors in the infertile population. There are very few studies investigating the process from the beginning of the process to the pregnancy outcome, which is the purpose of IVF treatment. In this context, it is thought that our study will contribute to the literature, which is limited in terms of monitoring the process from the beginning of infertility treatment to pregnancy determination. Although the relationship between psychological factors and infertility has been of interest to researchers from many disciplines, the number of studies examining the effect of these factors on pregnancy outcome is quite limited.

In addition, only unexplained infertile couples were included in the study and other factors affecting pregnancy outcome were excluded, and it was aimed to clearly evaluate the effect of anxiety and depression on pregnancy outcome.

## METHODS

### Design

This was a prospective cohort study.

### Sample and participants

The study data were collected between November 2017 and July 2018. The population of the study consisted of childless couples aged 18 years and older with a diagnosis of unexplained infertility who applied to Acıbadem Eskişehir Hospital IVF Center and volunteered to participate in the study. In determining the sample size, the G Power 3.1.9.2 program was used to calculate the sample size of the study. For the F-test analysis, it was found that a total of 76 people should be reached within the 95% confidence interval, taking into account the moderate effect size (η²=0.06) (80% statistical power, 0.05 alpha value). However, considering the possibility of losing the sample and to increase the power of the study, all couples who met the inclusion criteria were included in the study. In the study, it was aimed to reach the entire population and no sample was selected. However, 13 of these couples did not make it to the embryo transfer stage. In addition, five couples discontinued the treatment for non-medical reasons. Therefore, the study was completed with 102 couples, i.e., 102 women and 102 men.

### Data collection tools

The Personal Information Form, Case Follow-up Form, Spielberger State and Trait Anxiety Scale, and Beck Depression Inventory (BDI) were used to collect the data. The questionnaires were applied by the researcher through face-to-face interviews. The interview was completed separately in the interview room, first for women and then for men, so that the answers given by the couples would not be affected by each other.

### The Personal Information Form

The Personal Information Form consists of questions about the socio-demographic and familial characteristics of the couples and their infertility history.

### Case Follow-Up Form

It was prepared by the researchers to follow and record the treatment process. This form consists of information such as follicle follow-up, spermiogram results, number of oocytes obtained, time of HCG administration, oocyte pickup (OPU) and ET dates, and pregnancy outcome.

### Spielberger State-Trait Anxiety Inventory

The STAI-S (state) anxiety scale consists of 20 items, each ranging in score from 1 to 4. The STAI-S scale measures how participants feel “right now, at the moment.” The STAI-T (trait) anxiety scale also consists of 20 statements that measure how respondents generally feel. Each anxiety scale score varies from a minimum of 20 to a maximum of 80. The highest possible score is 80, and the lowest possible score is 20. High scores indicate high anxiety levels, and low scores indicate low anxiety levels. The scores are evaluated as follows: 0–19 points mean no anxiety; 20–39 points mean mild anxiety; 40–59 points mean moderate anxiety; 60–79 points mean high anxiety; and 80 points mean severe anxiety (panic)^6^.

### Beck Depression Inventory

The scale includes 21 questions scored between 0 and 3. The highest possible score is 63, and the lowest possible score is 0. The scores are evaluated as follows: 0–10 points mean no depression; 11–17 points mean mild depression; 18–29 points mean moderate depression; and 30–63 points mean severe depression^7^.

### Procedure

Before IVF, women’s serum levels of hormones such as follicle-stimulating hormone, estradiol, and luteinizing hormone were evaluated. Transvaginal ultrasonography and office hysteroscopy were also performed to evaluate uterus, ovaries, and cervix. A sperm sample was obtained from men in order to evaluate sperm parameters (e.g., count, motility, and morphology). Controlled ovarian hyperstimulation involving the use of fertility medications was used to induce ovulation by multiple ovarian follicles. During controlled ovarian hyperstimulation, serial ultrasound monitoring and serum estradiol measurements were performed in order to safely stimulate the ovaries. Oocyte retrieval was performed under general anesthesia using transvaginal ultrasound after obtaining required follicles. Semen samples were obtained at the time of oocyte retrieval, and sperm motility and morphological assessment were performed. The morphology of oocytes at the time of intra-cytoplasmic sperm injection (ICSI) was evaluated. Oocytes were microinjected by a spermatozoon. At 16–18 h after the ICSI procedure, a fertilization check was performed. Each woman received the antagonist short protocol, and day 5 embryos were transferred.

The procedure of the study included two phases. Data were collected from the couples at two different times: on the day of treatment starting and on the day of egg collection/semen sampling.

Phase 1: On the first day of treatment, the Personal Information Form, Spielberger State and Trait Anxiety Scale, and Beck Depression Inventory were administered separately to the man and woman. In the Case Follow-up Form, the necessary information about the KOH protocol, the status of the follicles, and the spermiogram results of the man was recorded, and this form was used by the researcher to follow and record the process until the end of the treatment.

Phase 2: Couples who came to the center for egg collection and semen sampling were administered the Spielberger State Anxiety Scale and Beck Depression Inventory just before the procedures. At the same time, the sperm sample data of the man for ICSI and the number of oocytes collected from the woman were obtained from the embryology laboratory and recorded on the Case Follow-up Form. In addition, at the last stage of the treatment, information such as embryo transfer and pregnancy results was obtained from the patient files and the last stage of data collection was completed. The study data were collected over a 9-month period between November 2017 and July 2018.

### Data analysis

The data were evaluated using the SPPS (Statistical Package for Social Science) 25.0 software. For comparisons between groups, the independent-sample t-test analysis was used for cases with two groups.

### Ethical considerations

Necessary approvals and permissions were obtained from the Acıbadem Mehmet Ali Aydınlar University Ethics Committee in Clinical Research (Decision No: 2017-ATADEK-16/12) and the IVF Center of Eskişehir Acıbadem Hospital (Decision No: 11/10/2017-1738).

## RESULTS

The mean age is 28.58±3.92 years for women and 31.72±3.91 years for men. Notably, 70.6% of the couples have a marriage period of “1–5 years,” 43.1% of the couples have been receiving infertility treatment for less than 1 year, and 85.3% have had their first IVF trial (Table 1).

**Table 1.** Sociodemographic and infertility history of couples.

Characteristics	Women (n=102)		Men (n=102)	
	n%		n%	
Age (years) Mean±SD	28.58±3.92		31.72±3.91	
Educational level				
Primary school	8	7.8	10	9.8
Middle school	20	19.6	10	9.8
High school	25	24.5	30	29.4
University	49	48	52	51
Employment status	44	43.1		
Employed			100	98
Unemployed	58	56.9	2	2
Duration of marriage*				
0–1 years	6	5.9	6	5.9
1–5 years	72	70.6	72	70.6
6–10 years	20	19.6	20	19.6
Over 11 years	24	3.9	24	3.9
Duration of ınfertility Treatment*				
0–1 years	44	43.1	44	43.1
1–2 years	33	32.4	33	32.4
3–5 years	24	23.5	24	23.5
6–10 years	1	1	1	1
Number of IVF trials*				
First	87	85.3	87	85.3
Second	12	11.8	12	11.8
Fourth	2	2	2	2
Over five	1	1	1	1

Frequency distribution of categorical data. *Common data for men and women.

There was no statistically significant relationship between STAI and BDI levels and oocyte count (p>0.05). There was a statistically significant relationship between STAI-T and sperm count, BDI level, and sperm motility (p<0.05, Table 2).

**Table 2.** Comparison of state-trait anxiety inventory and BDI levels with oocyte count, sperm count, and sperm motility.

Scales	Level	n	Oocyte count	t	p
			$\bar{\text{X}}$ ±SS		
	Mild (20–39)	64	15.73±8.36	10.10	0.273
STAI-S (pre-treatment )	Moderate (40–59)	38	13.7±9.89		
	Mild (20–39)	55	15.12±8.25	0.178	0.859
STAI-S (day of OPU)	Moderate (40–59)	47	14.80±9.83		
	Mild (20–39)	36	12.88±8.89	–1.71	0.090
STAI-T*	Moderate (40–59)	63	16.07±8.94		
	No	69	14.37±8.17	–0.98	0.328
BDI (pre-treatment)	Yes	33	16.24±10.46		
	No	75	14.20±8.12	–1.47	0.144
BDI (day of OPU)	Yes	27	17.14±10.86		
**Scales**	**Level**	**n**	**Sperm count (×10^6^)** **(Pre-treatment)**	**t**	**p**
			** $\bar{\text{X}}$ ±SS**		
	Mild (20–39)	65	28.20±14.10	1.21	0.228
STAI-S (pre-treatment )	Moderate (40–59)	37	24.37±17.24		
	Mild (20–39)	36	29.30±15.70	2.42	0.017
STAI-T	Moderate (40–59)	63	21.60±13.33		
	No	92	26.44±14.47	–0.73	–0.465
BDI (pre-treatment)	Yes	10	30.20±22.55		
	No	92	26.51±12.13	–1.47	0.144
BDI (day of OPU)	Yes	10	31.00±17.81	–1.05	0,293
**Scales**	**Level**	**n**	**Sperm count (×10^6^)** **(Day of OPU)**	**t**	**p**
			** $\bar{\text{X}}$ ±SS**		
	Mild (20–39)	68	27.76±12.76	0.91	0.365
STAI-S (day of OPU)	Moderate (40–59)	34	25.32±12.77		
	No	92	26.51±12.13	–1.05	0.293
BDI (day of OPU)	Yes	10	31.00±17.81		
**Scales**	**Level**	**n**	**Sperm motility (%)** **(Pre-treatment)**	**t**	**p**
			** $\bar{\text{X}}$ ±SS**		
	Mild (20–39)	65	66.24±12.79	1.078	0.284
STAI-S (pre-treatment )	Moderate (40–59)	37	63.37±13.12		
	Mild (20–39)	69	66.21±13.19	1.144	0.255
STAI-T	Moderate (40–59)	33	63.09±12.27		
	No	92	66.06±12.64	2.069	0.041
BDI (pre-treatment)	Yes	10	57.30±13.51		
**Scales**	**Level**	**n**	**Sperm motility (%)** **(day of OPU)**	**t**	**p**
			$\bar{\text{X}}$ ±SS		
	Mild (20–39)	68	68.79±10.93	0.456	0.649
STAI-S (day of OPU)	Moderate (40–59)	34	67.79±9.34		
	No	92	69.04±10.26	1.735	0.086
BDI (day of OPU)	Yes	10	63.10±10.57		

t: Student’s t-test. *As there were only three women whose trait anxiety scores were at the severe anxiety level, the mean number of oocytes of these women was excluded from the analysis.

The pregnancy rate of women with mild STAI level was higher than that of women with moderate level, and there was no statistically significant difference between them (p>0.05, Table 3). There was no statistically significant relationship between BID scores and the pregnancy rate (p>0.05, Table 3).

**Table 3.** Effect of state-trait anxiety inventory and BID level on pregnancy outcome.

STAI-S (pre-treatment)	Pregnant (n=60)		Not pregnant (n=42)		Total (n=102)	X2	p
	n	%	n	%			
Mild (20–39)	38	59.37	26	40,62	64	0.022	0.883
Moderate (40–59)	22	57.89	16	42.10	38		
**STAI-S (day of OPU)**	**Pregnant (n=60)**		**Not pregnant (n=42)**		**Total (n=102)**	**X2**	**p**
	**n**	**%**	**n**	**%**			
Mild (20–39)	33	60	22	40	55	0.068	0.794
Moderate (40–59)	27	57.44	20	42.55	47		
**Pregnant (n=57)**			**Not pregnant (n=42)**		**Total** **(n=99)**	**X2**	p*
**STAI-T**	**n**	**%**	**n**	**%**			
Mild (20–39)	22	61.11	14	38.88	36	0.289	0.591
Moderate (40–59)	35	55.55	28	44.44	63		
**BDI (pre-treatment)**	**Pregnant (n=60)**		**Not pregnant (n=42)**		**Total (n=102)**	**X2**	**p**
	**n**	**%**	**n**	**%**			
No	39	56.52	30	43.47	69	0.467	0.495
Yes	21	63.63	12	36.36	33		
**BDI (Day of OPU)**	**Pregnant (n=60)**		**Not Pregnant (n=42)**		**Total (n=102)**	**X2**	**p**
	**n**	**%**	**n**	**%**			
No	42	56	33	44	75	0.933	0.334
Yes	18	66.66	9	33.33	27		

X^2^: chi-square test. *As there were only three women with a STAI-T score of severe anxiety, these three individuals were excluded from the analysis.

## DISCUSSION

In the study, women with mild state anxiety had higher oocyte counts than women with moderate state anxiety, but there was no statistically significant difference between them. According to the Beck Depression Inventory mean scores, whether women were depressed before treatment and on the day of OPU did not affect the oocyte counts. There are many psychological factors other than anxiety and depression during IVF treatment. Some of these factors include a woman’s hopelessness, anger, loneliness, self-perception, body image, coping strategies, fears, and harmony with her partner. In addition, some factors such as lifestyle behaviors, eating habits, body mass index, and genetic and environmental factors are undeniable characteristics that may affect the number of oocytes. Xu et al., conducted a study with 842 primary infertile women to investigate the effects of anxiety and depression on IVF outcome and found no statistically significant difference in the number of oocytes in terms of anxiety or depression levels^8^. Although the number of studies examining the effect of anxiety and depression on oocyte count is limited, it supports the results of our study^9^. In the study, a significant difference was found between STAI-T level and sperm count and between BID level and sperm motility.

Bhongade et al., conducted a study with 70 infertile men in India to investigate the effect of psychological stress on seminal quality and found that anxiety and depression negatively affected sperm count, motility, and morphology^10^. In a study of 60 fertile and 112 subfertile men in Poland, Wdowiak et al., found that sperm count and semen volume were correlated with STAI-I, STAI-II, and BDI scores, and as a result, anxiety and depression were found to reduce semen volume and sperm density^11^. In the literature, the number of studies examining the effect of anxiety and depression on sperm count and motility is limited, and not many studies were found^9,12^.

In this study, women with mild anxiety had higher conception rates than women with moderate anxiety, but there was no statistically significant difference between them. Depression level had no effect on conception rates in the study. The results of studies examining the effect of anxiety and depression on pregnancy outcome differ from each other. Although the majority of studies suggest that these psychological elements do not affect pregnancy, there are also studies that find a significant relationship between anxiety and depression and pregnancy outcome^13-16^. Haimovici et al., found that stress, anxiety, and depression affected pregnancy success rates^13^. Kalaitzaki et al., confirmed the negative impact of stress on IVF outcome^14^. The results of these studies show differences with our study. Considering that the infertility diagnosis and history of the women in the sample group are different, the time of measurement varies, and there may be many other factors affecting pregnancy success, it is inevitable that the results will be different.

There are also studies in the literature that did not find a significant relationship between anxiety and depression and pregnancy outcome^1,12,17-25^. In their study conducted in the Netherlands with 690 infertile women undergoing IVF treatment, Lintsen et al., found no relationship between state anxiety and depression and pregnancy outcome^22^. In a study conducted in China with 264 women, An et al., found no significant relationship between state anxiety and depression both before treatment and on the day of OPU and pregnancy outcome^21^. In a study conducted in China with 107 women receiving IVF treatment for the first time, Li et al., found no significant relationship between pre-treatment and OPU day state anxiety and depression and pregnancy outcome^20^. Zaig et al., conducted a study with 108 women in Israel and found no significant relationship between state anxiety and depression scores during ovulation induction and pregnancy outcome^23^. Pasch et al., conducted a study with 200 primary and secondary infertile women in San Francisco to examine the effect of psychological stress on IVF treatment and found no effect of pre-treatment depression scale scores on pregnancy outcome^26^. Hashemi et al., administered the STAI-S and STAI-T scales to 180 women in Tehran at the last control before the OPU day to investigate the relationship between anxiety and IVF success^18^. In the study, no significant difference was found between STAI-S and STAI-T levels and pregnancy outcome.

In a study conducted in Hong Kong with 360 infertile women, Saravelos et al., found no significant relationship between state anxiety and depression scores on the day of OPU and pregnancy^24^. Bapayeva et al.’s statistical analysis showed that IVF outcome was not significantly associated with depression and stress, while higher anxiety scale scores were negatively associated with clinical pregnancy after IVF^1^. According to Liu et al., the anxiety and depression scores of infertile couples in different stages were not related to the outcome of IVF-ET^3^. According to Pacivic et al., anxiety and depression symptoms did not prove to be statistically significant predictors of the IVF outcome^5^. The findings reported in our study support the results in the literature.

## CONCLUSION

The study shows that anxiety and depression are not significantly correlated with the outcome of IVF. The impact of anxiety/depression on IVF outcome remains unclear. Infertility and IVF treatment is a complex process with many dimensions and multiple factors. The relationship between psychological factors and successful IVF outcome is more complex than is commonly believed. Identification of risk and protective psychological factors may contribute to increased pregnancy rates and encourage the implementation of specific therapeutic interventions. However, more evidence-based studies that examine multiple factors are needed.
